# Hospital Out-Patient Treatment of Infants

**Published:** 1906-12-29

**Authors:** J. Walter Carr

**Affiliations:** Physician to the Royal Free Hospital and to the Victoria Hospital for Children, Chelsea


### Hospital Clinics.

### HOSPITAL OUT-PATIENT TREATMENT OF INFANTS.

By J. WALTER CARR, M.D.Lond., F.R.C.P.Lond., F.R.C.S.Eng., Physician to the Royal Free Hospital and to the Victoria Hospital for Children, Chelsea.

The question of reform of the out-patient department of hospitals is in the air. At the United Hospitals Conference at University College, on December 6, Dr. Heron, amid the sympathetic cheers of his audience, denounced the out-patient treatment of consumptives as a farce, and that of chronic dyspeptics as practically useless. That his statements were none too strong will be acknowledged by all competent to judge; but there are also other classes of out-patients which call for consideration, and the question may well be raised whether the out-patient treatment of the diseases of infants, especially in connection with our children's hospitals, is as satisfactory as it might and should be.

The all-important fact from which to start is that improper feeding is by far the greatest cause of infantile morbidity and of infantile mortality. Of the total number of patients attending the medical out-patient department of a children's hospital, probably fully one-third are under two years of age, and nothing is more striking than to note what a small proportion of these infants have been properly fed. Summer diarrhœa is the scourge of the infantile population of our large cities, giving rise during the hot months of the year to more deaths than all the other zymotic diseases put together, and it is the most destructive of all the ailments incidental to child life. Last August the deaths from it in London alone rose to about 500 a week. Were only one-half of this number of deaths to result from small-pox or scarlet fever, the matter would at once be regarded as of first-class national importance; but because it is merely a question of diarrhœa, no one troubles. Yet this enormous mortality is eminently preventable, for a fatal case of summer diarrhœa in an infant fed exclusively at the breast is comparatively rare. Then we have rickets (with which may be mentioned infantile scurvy), an exceedingly common disease and with far-reaching consequences to almost every organ of the body, due almost entirely to improper food, and often to food which is relatively expensive. Moreover, the influence of improper feeding is in many ways far wider than is commonly appreciated: to take but a single instance, how many people associate bronchitis with unsuitable food? Yet the great majority of young children who suffer from severe bronchitis or from broncho-pneumonia (most common diseases in the winter months) are either rachitic or marasmic; in other words, they have been improperly fed; their vitality and resistive power have been lowered: consequently they are predisposed to bronchitis and are more likely than previously healthy children to succumb to it once it has developed. Nor if these badly fed children survive do their troubles necessarily end with infancy; only too often they grow up stunted and ill-developed, prone to diseases of all kinds, especially perhaps to tubercle, and likely to transmit to their children a feeble physique and deficient resistive power.

How do children's hospitals deal with all these evils? At present, unfortunately, they only meet them too late, when the mischief is fully developed, when the results of improper feeding can at best only be palliated and cannot possibly be cured. Yet pre-eminently in dealing with children, prevention rather than cure is urgently needed.

Under existing conditions the child is only too often first seen at the hospital after he has been weaned, after the improvised methods of feeding of the mother and of her neighbours have utterly failed, when he is already profoundly rachitic or marasmic, or is perhaps well nigh moribund from diarrhœa. It is being increasingly recognised, therefore, that, first and foremost, we need some plan to encourage breast-feeding during the early months of infancy. It is a significant fact that, whilst the mortality generally throughout the country has been steadily falling for many years past, the infantile death-rate has been well nigh stationary. The explanation is that improvements in general hygiene have been counter-balanced by a steady diminution in breast-feeding. That an increasing number of mothers in our large cities are really unable to suckle their children is undoubted, but it is equally true that a still larger number wantonly evade their responsibility; whilst the specious and omnipresent advertisements of innumerable infant foods, wickedly suggesting that perfect substitutes for human milk are readily and cheaply obtainable, doubtless help to induce many mothers to wean their children on the slightest excuse. Dr. Sykes, Medical Officer of Health for St. Pancras, has lately been devoting special attention to the development of systematised efforts to encourage mothers to suckle their babies, and it is most interesting to note that in that borough last summer there was a marked decrease in the infantile mortality from diarrhœa, as compared with recent years; whereas in other parts of London, and throughout the country generally, the mortality, owing to the prolonged hot weather, was considerably higher than usual. No adequate explanation for this uniqu[illegible]rovement in St. Pancras can be suggested except increased breast-feeding.

Our steadily diminishing birth-rate increases the importance of this matter, and we need to organise some such system as that already developed in France under the name of "Consultations de Nourrissons," or like that started in St. Pancras by Dr. Sykes, the primary object of which shall be to encourage mothers by every means to nurse their children. In addition, further arrangements are required for watching children whom the mothers are really unable to nurse, and also those who have been weaned after the normal period of lactation; for many a baby, healthy enough when weaned at the age of nine or ten months, suffers grievously from being almost at once fed on what is roughly characterised by the mother as "just what we have." The hospital medical officers see the child first when the mischief is already done, and can only give good advice as to the future feeding—advice which, even when not received too late, is in a large number of cases, partly from ignorance, partly from poverty, and partly, it may be, from sheer indifference, largely or completely ignored. The child needs to be followed to its home by some organisation analogous to that which Dr. Philip has established in Edinburgh for the treatment and supervision of tuberculous patients in their own homes, through the agency of the Tuberculosis Dispensary (*vide* The Hospital for December 8, 1906). In connection with such an institution really pure and, if necessary, suitably modified milk should be provided, to be sold at a reasonable price to mothers able to pay for it, and perhaps under cost price (with proper precautions, possibly under the supervision of the Poor-law authorities) to the genuinely poor. If by such methods mothers could be encouraged to nurse their children, could receive prompt and effective guidance in dealing with difficulties in continuing lactation, and in the proper methods of feeding after weaning, the hospital out-patient department remaining as a resort where further advice or actual medical treatment could be obtained when necessary, then the work of these departments in children's hospitals, and to a large extent also in general hospitals, would be greatly diminished, and at the same time rendered far more effective than at present.

